# Dyadic Coping, Respiratory Sinus Arrhythmia, and Depressive Symptoms Among Parents of Preschool Children

**DOI:** 10.3389/fpsyg.2018.01959

**Published:** 2018-10-16

**Authors:** Andrew Switzer, Warren Caldwell, Chelsea da Estrela, Erin T. Barker, Jean-Philippe Gouin

**Affiliations:** Department of Psychology, Concordia University, Montreal, QC, Canada

**Keywords:** dyadic coping, respiratory sinus arrhythmia, heart rate variability, depression, stress, social support

## Abstract

Respiratory sinus arrhythmia (RSA) is a biomarker of cardiac vagal tone that has been linked to social functioning. Recent studies suggest that RSA moderates the impact of interpersonal processes on psychosocial adjustment. The goal of this study was to assess whether RSA would moderate the association between dyadic coping (DC) and depressive symptoms. Eighty cohabiting couples raising preschool children completed the Dyadic Coping Inventory, the Center for Epidemiological Study-Depression scale and had their RSA assessed during a laboratory session. Couples completed follow-up assessments of depressive symptoms 6 and 12 months later. Data were analyzed using an Actor-Partner Interdependence Model. Results indicated that RSA moderated the actor effect of negative DC on depression in men, such that men with lower RSA had a stronger association between their own ratings of negative DC within the couple relationship and their own depressive symptoms, compared to their counterparts with higher RSA. RSA also moderated the partner effect of delegated DC on depressive symptoms. Among men with higher RSA, there was a significant negative association between their partner’s ratings of delegated DC within the couple relationship and the men’s depressive symptoms, whereas partner-rated delegated DC was unrelated to depressive symptoms among men with lower RSA. These results suggest that men with higher RSA may possess social skills and abilities that attenuate the association between stressful marital interactions and negative mood.

## Introduction

Stress and coping research has traditionally focused on how individuals react to and are impacted by stress from an individual perspective. The systemic-transactional model of dyadic coping (DC) highlights that for couples, coping occurs in a shared social context characterized by the interdependence of partners’ responses to stress (Bodenmann, 1995). In this context, coping is often a dyadic rather than individual endeavor, whereby members of a couple work *together* to cope with stress. According to this model of DC, when one or both members of a dyad experience stress, both partners engage in a series of reciprocal interactions following an initial communication of stress. These interactions form a DC framework, which can take many forms. Supportive DC refers to strategies that assist one’s partner in coping with his/her stressors (e.g., providing advice, empathy, etc.). Delegated DC refers to one partner relieving his/her partner of other responsibilities (e.g., housekeeping, shopping, etc.). Common DC refers to joint efforts of a couple to cope with a stressor directly (e.g., joint problem solving, sharing and seeking information together, etc.). These three types of DC are considered positive forms of DC. However, as with individual coping efforts, not all DC responses are positive. Negative DC includes reacting to a partner’s stress communication with indifference, ambivalence, or hostility. The systemic-transactional model stipulates that engagement in positive DC fosters both better adjustment to stress as well as enhanced relationship satisfaction.

A large body of research has reported the benefits of positive DC for relationship functioning. A recent meta-analysis from Falconier et al. (2015) reported a moderate positive correlation (*r* = 0.45) between a couple’s positive DC and relationship satisfaction. This association was observed across research methodologies (e.g., cross-sectional, longitudinal, experimental/intervention, different measurement tools), sample characteristics (e.g., culture, SES, education, age, gender), and types of stressors (e.g., chronic illness, child-related stress, etc.). Overall, more positive DC and less negative DC consistently predicted greater relationship satisfaction.

Although DC has been reliably associated with relationship satisfaction, its association with psychological adjustment is less consistent. Nevertheless, positive DC has been associated with improved psychological adjustment in different contexts. Greater positive and common DC predicted less depression in community samples of cohabiting couples (Bodenmann et al., 2011; Gana et al., 2017). Supportive DC moderated the link between discrimination stress and depression among same-sex couples (Randall et al., 2017). Positive DC also appears to promote better adjustment to chronic medical illness: in cancer patients, greater supportive and common DC have been associated with less depression (Badr et al., 2010; Regan et al., 2014; Ernst et al., 2017). Similar results have been found among couples of patients with diabetes, chronic obstructive pulmonary disease, and dementia (Meier et al., 2011; Gellert et al., 2017; Zajdel et al., 2018). Furthermore, greater supportive DC was associated with less parenting stress among parents of children with autism (García-López et al., 2016).

There are, however, some inconsistencies in this literature. The specific forms of DC associated with individual adjustment differ across studies. Rottmann et al. (2015) reported a significant association between common DC and psychological adjustment, but not with supportive DC. Conversely, Gellert et al. (2017) reported significant associations between supportive DC and delegated DC with psychological adjustment, but not with common DC. Furthermore, positive DC has been associated with increased depression and anxiety in certain contexts. For example, cancer patients who reported engaging in more delegated DC had worsened depression (Rottmann et al., 2015). Similarly, among patients with chronic obstructive pulmonary disease, greater delegated DC was associated with poorer quality of life in some (Meier et al., 2011), but not in all studies (Vaske et al., 2015). Moreover, some researchers have reported no significant association between positive DC and depression (Feldman and Broussard, 2006; Breitenstein et al., 2012).

In contrast to positive DC, negative DC has been more consistently related to poor psychological adjustment. When an individual feels that their communication of stress is being met with indifference, ambivalence, or hostility, they tend to report worsened psychological adjustment. Among couples from the community, greater negative DC was related to higher anxiety and depression (Bodenmann et al., 2011; Karademas and Roussi, 2016). Similarly, negative DC was associated with worsened psychological adjustment among couples facing cancer (Badr et al., 2010; Regan et al., 2014; Rottmann et al., 2015). However, some inconsistencies have also been reported. Gellert et al. (2017) reported no significant relationship between negative DC and depression.

The inconsistencies in the associations between DC and psychological adjustment suggest that individual or situational factors may moderate the impact of DC on individual adjustment. Indeed, there is evidence that not everyone benefits equally from spousal support (Vella et al., 2008; Meuwly et al., 2012; Kordahji et al., 2015). Furthermore, it is likely that not all individuals possess the social skills and abilities required to successfully enact the DC process. DC is an interactional process that involves stress communication by one partner, perception of stress by the other partner, followed by the partner’s coping reaction to the stress communication (Bodenmann, 2005). These three components of the transactional cycle require specific skills for effective DC (Bodenmann and Randall, 2012). Individuals who lack the ability to effectively communicate their needs, to recognize their partner’s distress, or to regulate their own negative emotions in order to provide effective support may have difficulty enacting an optimal DC process (Bodenmann et al., 2004; Gabriel et al., 2016; Levesque et al., 2017). Individual variations in social engagement capacities may thus moderate the impact of DC on psychological distress.

Respiratory sinus arrhythmia (RSA) is a measure of cardiac vagal tone that has been conceptualized as a biomarker of social engagement capacities (Porges, 2003a, 2007). RSA is calculated from the fluctuations in time intervals between consecutive heartbeats linked to the respiration cycle. At rest, the combined actions of the sympathetic and parasympathetic branches of the autonomic nervous system regulate cardiac activity. Vagal-dependent parasympathetic output provides tonic and fast-acting inhibitory influences on cardiac activity that are temporarily lifted during the inspiration phase of the respiration cycle, leading to rapid fluctuations (0.5 s) in interbeat intervals. In contrast, sympathetic stimulation occurs on a longer timeframe over the course of 1–4 s. Accordingly, RSA, or high-frequency heart rate variability, representing fluctuations in beat-to-beat time intervals, indexes mostly vagally mediated parasympathetic output to the sino-atrial node of the heart (Berntson et al., 1997).

Two major theoretical frameworks link RSA with social functioning. Porges’ polyvagal theory proposes that the mammalian autonomic nervous system evolved to support social engagement behavior (Porges, 2003a, 2007). The development of parasympathetic modulation of cardiac activity through the vagal nerve allowed for rapid shifts in energy mobilization that facilitated the emergence of social engagement behaviors in response to stress, instead of the more metabolically costly fight-or-flight response (Porges, 2003b). This theory also suggests that throughout vertebrate evolution, structural and functional connections emerged among brain stem nuclei involved in the neural control of cardiac activity, the striated muscles of the face, and the smooth muscles of the viscera. In more evolved mammals, the brain stem nuclei regulating heart rate activity became connected to the soft palate, pharynx, larynx, eyelid, middle ear, and other facial muscles involved in emotional expression and social communication behaviors, allowing for the coordination of physiological and behavioral states supporting social engagement responses (Porges, 2003b). The vagus nerve, linking peripheral physiology and central functions, plays a key role in quickly shifting autonomic states to modulate the repertoire of social and behavioral responses that can be expressed at a given time (Porges, 2003b). RSA is then conceptualized as a biomarker of the neurophysiological system supporting social engagement behavior (Porges, 2007). According to this theory, individuals with lower RSA are more likely to exhibit compromised spontaneous social behavior, social awareness, and emotional expressivity, and they are less physiologically regulated by positive social interactions.

The neurovisceral integration model also proposes that modulation of physiological arousal via the vagal nerve allows rapid and flexible responses to changing environmental demands. This model highlights the neural connections among the vagal nerve and cortical and subcortical brain structures that modulate the inhibitory processes regulating peripheral physiological arousal. Neuroimaging studies indicate that RSA is associated with ventromedial prefrontal cortex, anterior cingulate and amygdala activity, with greater prefrontal cortex activity being linked to higher RSA (Thayer et al., 2012). RSA is thus conceptualized as a physiological marker of top-down neural processes involved in self-regulatory capacity (Thayer and Lane, 2009). In particular, it has been argued that individuals with higher RSA may have better emotion regulation capacities that in turn allow them to maintain high relationship quality despite elevated stress (Diamond et al., 2011).

Tonic or resting RSA has been related to various markers of social functioning. Higher RSA has been associated with more prosocial behavior (Beauchaine et al., 2013), better emotion recognition (Quintana et al., 2012), better empathic accuracy (Côté et al., 2011), less self-reported alexithymia (Lischke et al., 2018), greater compassion (Stellar et al., 2015), less hostility (Sloan et al., 2001), greater attachment security (Diamond and Hicks, 2005; Maunder et al., 2012), better acculturation (Doucerain et al., 2016), and more positive marital functioning (Diamond et al., 2011; Smith et al., 2011; Donoho et al., 2015). RSA has also moderated affective responses to social interactions. Higher resting RSA was associated with a stronger association between social events and positive affect (Isgett et al., 2017). Among dating couples, women with higher resting RSA showed a larger within-person association between their partner-reported positive couple interactions and their own positive affect, compared to women with lower RSA (Diamond et al., 2011). Individuals with higher resting RSA also exhibited a stronger association between high social support and fewer depressive symptoms over time (Hopp et al., 2013). RSA also moderated the association between maternal depression and expression of negative emotions during a mother-adolescent dyad conflict discussion (Connell et al., 2011). Collectively, these findings provide indirect evidence that RSA may influence the extent to which an individual can benefit from the DC process.

### The Present Study

The primary goal of this study was to assess whether RSA would moderate the association between DC and depressive symptoms. Given that the impact of DC on psychological adjustment may be more salient during a period of increased stress (Cohen and Wills, 1985), this study was conducted among parents of young children, a normative developmental period associated increased with psychosocial stress (Umberson et al., 2010). Indeed, parents of preschool children are more likely to feel overwhelmed by the daily demands and time constraints of caring for young children, and to experience straining work-family conflict compared to parents of older children (Scharlach, 2001; Nomaguchi and Milkie, 2003). Furthermore, the transition to parenthood is also associated with increased marital conflict and decreased marital satisfaction that may last at least until the children reach school years (Crohan, 1996; Keizer and Schenk, 2012). Given this increased exposure to psychosocial stressors, it is not surprising that a significant percentage of parents of young children experience elevated depressive symptoms that often last throughout the preschool years (Evenson and Simon, 2005; Horwitz et al., 2009; Garfield et al., 2014). This normative developmental period represents a period of time where the role of DC may be especially important in helping individuals adjust to the daily parenting challenges that the couple is facing (García-López et al., 2016; Zemp et al., 2017). In this context, the role of RSA in modulating the effect of DC may be salient. We hypothesized that individuals with higher RSA might benefit more from DC than their counterparts with lower RSA.

## Materials and Methods

### Participants

Couples were invited to participate in a study of parenting stress among parents of preschool children. Participants were recruited via online advertisements as well as through schools and support groups for parents of children with developmental disabilities. Parents of children with neurodevelopmental disorders or disabilities (e.g., autism spectrum disorder, intellectual disability, cerebral palsy) were oversampled (21.3% of dyads), because these parents tend to experience more parenting stress and greater psychological distress than parents of typically developing children (Hayes and Watson, 2013), thereby increasing the range of parenting challenges within the sample. To be included in the current study, cohabiting couples were required to be the legal guardian of a child under the age of 7. Exclusion criteria included pregnancy, breastfeeding, chronic medical conditions, and regular prescribed medication use. These exclusion criteria aimed at minimizing external factors that may impact RSA.

The full sample included 84 heterosexual couples. However, one couple did not complete the DC assessment, for two other couples only one partner completed the DC assessment, and one participant did not complete the depression assessment. The final sample used for the actor-partner interdependence analyses thus included 80 couples. Participants had a mean age of 34.60 (*SD* = 4.70) years, ranging from 21 to 48, and their children had a mean age of 36 (*SD* = 22.74) months, ranging from 5 to 89. In this ethnically diverse sample, 55.36% of participants identified as Caucasians. About 36.9% of participants had completed a high school degree or lower level of education, 41.7% had completed a technical degree, and 21.5% a university degree. The average household income was $55,000 (*SD* = $8900) CAD. About 56.5% of the participants were employed full time, 17.9% were working part-time, and 25.6% were not currently working. Couples had been cohabiting for an average of 8.11 (*SD* = 3.42) years. Most couples had either one (44%) or two (48%) children.

### Procedure

Couples first completed online self-report questionnaires assessing DC and depressive symptoms. Subsequently, they completed a 60-min laboratory visit to assess RSA. During the laboratory visit, couples were seated side-by-side in comfortable chairs and fitted with snap electrodes in a lead II configuration for electrocardiogram (ECG) recording. They participated in several tasks: a 5-min seated and silent resting period where participants were instructed to “breathe normally and relax as much as possible without falling asleep” a 5-min questionnaire about their child’s behavior problems was completed independently by each parent; a marital interaction task, in which each dyad member was instructed to take turns leading a 7-min discussion about “the most difficult aspect of raising young children and how it has impacted your relationship with your partner,” as well as how they would like their partner “to change regarding the way they raise your child,” and a 5-min silent and seated recovery period. Participants remained seated throughout the tasks. A retractable curtain separated the partners during the resting baseline and recovery periods in order to prevent them from interacting with each other during these time periods. An experimenter monitored the couples during the experimental tasks via a control room and prompted them to comply to the instructions when couples deviated from the protocol.

All participants were asked to refrain from consuming caffeine (France and Ditto, 1992), alcohol (Weise et al., 1986), tobacco (Hayano et al., 1990) or engaging in vigorous exercise (Houtveen et al., 2002) in the 2 h prior to the laboratory session. After the laboratory visit, both members of the couple independently completed a daily diary for 6 consecutive days to assess daily stress. Depressive symptoms were re-assessed using online questionnaires sent via email to participants 6- and 12-months after the laboratory visit. This study was approved by Concordia Human Research Ethics Committee. Each member of the couples provided written informed consent prior to participation. Each couple received $100 CAD following the completion of the study.

### Measures

Depressive symptoms were assessed using the Center for Epidemiological Study-Depression scale (CES-D). The CES-D assesses the frequency of various depressive symptoms in the past week (e.g., restless sleep, poor appetite, and feeling lonely). Cronbach’s α was 0.90 in this sample. The CES-D was administered before the laboratory visit, and 6- and 12-months following the visit. Higher scores indicated more depressive symptoms.

DC was assessed using the Dyadic Coping Inventory (DCI; Bodenmann, 2008). The DCI measures DC responses to stress enacted by oneself, by one’s partner, as well as the couple’s joint coping efforts using a 5-point Likert scale. In the present study, supportive (Cronbach’s α = 0.76), delegated (Cronbach’s α = 0.63), negative (Cronbach’s α = 0.71) and common DC (Cronbach’s α = 0.86) were assessed. Perception of DC by oneself and one’s partner were summed for the supportive, delegated, and negative DC subscales. Higher scores indicated more DC from both members of the dyad.

Daily stress was assessed in a daily diary format using three items adapted from the Perceived Stress Scale (Cohen et al., 1983). At the end of each day for 6 consecutive days after the laboratory visit, participants reported to what extent they felt: (1) “that difficulties were piling up”; (2) “overwhelmed”; (3) “that they were able to control important things in their life” (reverse coded), on a 5-point Likert scale ranging from “not at all” to “a great deal.” These ratings were combined to create a daily stress measure (Cronbach’s α = 0.63) that was subsequently averaged across days to obtain an overall measure of stress for each participant.

Respiratory sinus arrhythmia was measured as part of a 60-min recording protocol. Data were collected using an ECG amplifier module within a Mindware BioNex 8-slot chassis (Mindware Technologies Ltd., Gahanna, OH, United States). Interbeat intervals were recorded continuously using a sampling rate of 1000 Hz. The ECG recordings were analyzed using MindWare RSA Analysis software, Version 3.1 (Mindware Technologies LTD., Gahanna, OH, United States). Recording artifacts were identified using an automated algorithm, and were visually inspected and corrected when necessary. Less than 1% of beats were edited for each participant. RSA was extracted using a Fast Fourier Transform to compute the natural log of the 0.15–0.40 Hz frequency band in order to isolate vagal-dependent parasympathetic influences on the heart. RSA was calculated by averaging the RSA value for each 30-s epoch across each task. The average RSA level across all tasks was used as an overall marker of vagal tone.

### Statistical Analyses

First, Spearman Rho’s correlations evaluated bivariate associations among actor depression, actor and partner DC, and actor RSA. An actor-partner interdependence model (APIM; Kenny et al., 2006) using multilevel modeling estimated associations between actor- and partner-rated DC and depressive symptoms, as well as the moderating impact of RSA and gender. The APIM model allowed for the simultaneous assessment of actor effects (e.g., the association between the wife’s own ratings of DC within the couple relationship and the wife’s own ratings of depressive symptoms) as well as partner effects (e.g., the association between the husband’s rating of DC within the couple relationship and the wife’s own rating of depression symptoms), while accounting for the within-couple dependency in the data structure. Preliminary analyses indicated that there was a significant increase in depressive symptoms for men over time, *β (SE)* = 0.18 (0.07), *t =* 2.73, *p* = 0.007, but not for women, *β (SE)* = 0.07 (0.09), *t* = 0.79, *p* = 0.43. Given the lack of change in depressive symptoms over time for women, the averaged depression scores across each of the three time points for each partner were used as the dependent variable in order to model the effects of DC on depressive symptoms simultaneously for men and women. In the current sample, the dyads were distinguishable (i.e., each dyad included a male and female partner). We used the two-intercept approach in order to simultaneously calculate separate equations for men and women (Kenny et al., 2006). A heterogeneous compound symmetry (CSH) covariance structure allowed for the estimation of unique variances of each dyad member. The moderating effect of RSA on the association between DC and depressive symptoms was tested using two-way interactions between RSA and DC. Following statistically significant interactions, simple slopes analyses were conducted by plotting the change in strength of the relationship between DC and depressive symptoms at two levels of the moderator, RSA (1 SD above and -1 SD below the mean).

All continuous variables were centered. RSA was normally distributed, but depressive symptoms showed a positively skewed distribution, which was corrected using a base 10 logarithmic transformation. The pattern of results did not change substantially when transformed variables were used. Analyses were run with transformed data, but untransformed analyses were plotted for greater interpretability. Given the inconsistencies in the associations between the different forms of DC and depression, separate models were run for each type of DC (Rottmann et al., 2015; Gellert et al., 2017). Having a child with a neurodevelopmental disorder was included as a covariate in each model given that these parents usually report greater psychological distress than parents of typically developing children (Olsson and Hwang, 2001). An alpha level of 0.05 was used for the present study. SAS PROC MIXED was used to perform multilevel modeling with restricted maximum likelihood estimation.

## Results

Correlations among the main study variables are presented in **Table 1**. Depressive symptoms were significantly positively correlated between cohabiting partners with a small effect size, *r* = 0.26, *p* = 0.03. Actor- and partner-ratings of DC were also positively correlated with medium effect sizes, ranging from *r* = 0.34 to *r* = 0.57. In contrast, there was no statistically significant correlation between partners’ RSA, *r* = 0.08, *p* = 0.48. In this sample, there were no statistically significant gender differences in depressive symptoms, *t* = 1.54, *p* = 0.13, or DC, all *p*’s > 0.25. However, RSA was significantly higher among women than among men, *t* = 2.68, *p* = 0.008.

**Table 1:** Spearman Rho’s correlations among the study variables.

	1	2	3	4	5	6	7	8	9	10
(1) Actor Depression	–	-0.39**	-0.31**	-0.37**	0.54**	-0.43**	-0.21	-0.34**	0.43**	-0.02
(2) Actor Supportive DC	-0.31**	–	0.66**	0.80**	-0.61**	0.54**	0.43**	0.53**	-0.48**	0.12
(3) Actor Delegated DC	-0.22**	0.58**	–	0.57**	-0.46**	0.41**	0.34**	0.35**	-0.22	0.06
(4) Actor Common DC	-0.23*	0.84**	0.50**	–	-0.50**	0.54**	0.44**	0.62**	-0.33**	0.07
(5) Actor Negative DC	0.31**	-0.68**	-0.38**	-0.62**	–	-0.37**	-0.28*	-0.33**	0.44	-0.001
(6) Partner Supportive DC	-0.25*	0.54**	0.43**	0.53**	-0.48**	–	0.58**	0.84**	-0.68**	0.15
(7) Partner Delegated DC	-0.21	0.41**	0.34**	0.35**	-0.22	0.66**	–	0.50**	-0.38**	0.30**
(8) Partner Common DC	-0.30**	0.54**	0.44**	0.62**	-0.34**	0.80**	0.57**	–	-0.62	0.04
(9) Partner Negative DC	0.17	-0.37**	-0.28*	-0.33**	0.44**	-0.61**	-0.47**	-0.51**	–	-0.09
(10) Actor RSA	-0.08	0.04	0.08	-0.08	0.005	-0.09	-0.02	-0.18	0.05	–
*M* (*SD*) women	17.96 (11.67)	36.91 (7.06)	14.04 (3.19)	23.72 (5.91)	16.98 (5.83)	36.00 (7.31)	13.58 (2.80)	23.41 (5.67)	15.91 (5.99)	6.53 (0.81)
*M* (*SD*) men	15.18 (8.84)	36.00 (7.31)	13.58 (2.80)	23.41 (5.67)	15.91 (5.99)	36.91 (7.06)	14.04 (3.19)	23.72 (5.91)	16.98 (5.83)	6.08 (0.99)

*Correlations for men are presented above the diagonal and correlations for women are presented below the diagonal. Actor effects represent the person’s own rating of DC with the couple relationship, whereas the partner effects represent the spouse rating of DC with the couple relationship. ^∗^*p* < 0.05; ^∗∗^*p* < 0.01*.

A series of models tested whether there were significant actor and partner effects of DC on depressive symptoms. Moderation effects were also tested using two-way interaction terms between actor- and partner-rated DC and actor RSA. In these models, being a parent of a child with a neurodevelopmental disorder and daily stress were added as covariates. Mothers of a child with a neurodevelopmental disorder reported significantly more depressive symptoms than mothers of a typically developing child, *β(SE)* = 0.22 (0.07), *t* = 3.35, *p* = 0.001, whereas fathers of a child with a neurodevelopmental disorder had marginally higher depressive symptoms, compared to fathers of a typically developing child, *β (SE)* = 0.11 (0.06), *t* = 1.80, *p* = 0.08.

**FIGURE 1:**
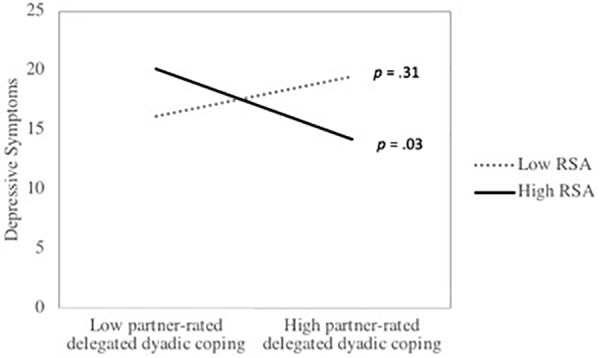
The association between partner-rated delegated DC and depressive symptoms as a function of respiratory sinus arrhythmia (RSA*)* among men. *p*-values represent the significance of the simple slopes at high (1 SD above the mean) and low (1 SD below the mean) of RSA.

**FIGURE 2:**
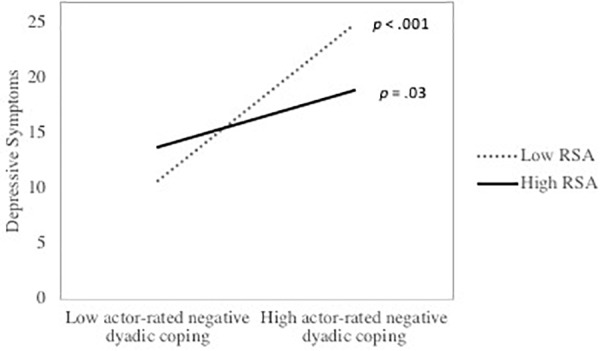
The association between actor-rated negative DC and depressive symptoms as a function of respiratory sinus arrhythmia (RSA) among men. *p* values represent the significance of the simple slopes at high (1 SD above the mean) and low (1 SD below the mean) of RSA.

**Table 2** presents the actor and partner DC effects as well as their interactions with actor’s RSA for men. Results indicated that, for men, there were significant actor effects of delegated DC and negative DC as well as a marginally significant actor effect of supportive DC. Significant partner effects of supportive DC and negative DC were also observed. Greater supportive and delegated DC were associated with less depressive symptoms, whereas more negative DC was related to greater depressive symptoms. There was also a marginally significant effect of daily stress, with greater daily stress being marginally associated with more depressive symptoms. Furthermore, actor’s RSA significantly moderated the effect of partner-rated delegated DC on depressive symptoms. Simple slope analyses indicated that there was a significant negative association between partner-rated delegated DC and actor’s depression among men with higher RSA but a non-significant positive association between the two constructs among participants with lower RSA. The interaction between partner-rated delegated DC and depressive symptoms is depicted in **Figure 1**. Furthermore, there was a significant interaction between actor-rated negative DC and actor RSA. Simple slope analyses indicated that the association between actor-rated negative DC and actor’s depression was stronger among participants with lower RSA than among individuals with higher RSA. **Figure 2** illustrates the interaction between actor-rated negative DC and actor’s RSA predicting actor’s depressive symptoms.

**Table 2:** Actor and partner effects of DC on depression and moderation effects of RSA for men.

	*β* (SE)	*t*	*p*	95% CI
***Supportive DC***			
Actor DC	-007 (0.004)	-1.96	0.05^τ^	[-0.01,0.0001]
Partner DC	-0.009 (0.004)	-2.05	0.04^∗^	[-0.02,-0.0002]
Daily Stress	0.01 (0.007)	1.94	0.06^τ^	[-0.003,0.03]
RSA	0.07 (0.13)	1.42	0.16	[-0.10,0.60]
Actor DC × RSA	0.001 (0.004)	0.44	0.66	[-0.007,0.01]
Partner DC × RSA	-0.003 (0.004)	-1.28	0.20	[-0.01,0.003]
Daily Stress × RSA	-0.008 (0.007)	-1.14	0.26	[-0.02,0.006]
***Delegated DC***			
Actor DC	-0.03 (0.01)	-2.85	0.006^∗^	[-0.05,-0.01]
Partner DC	-0.005 (0.009)	-0.56	0.57	[-0.02,0.02]
Daily Stress	0.01 (0.007)	1.76	0.08^τ^	[-0.002,0.03]
RSA	0.38 (0.17)	2.16	0.03^∗^	[0.03,0.72]
Actor DC × RSA	-0.0003 (0.01)	-0.03	0.97	[-0.01,0.02]
Partner DC × RSA	-0.02 (0.009)	-2.10	0.04^∗^	[-0.04,-0.001]
Daily Stress × RSA	-0.007 (0.007)	-0.91	0.37	[-0.02,0.008]
***Common DC***			
Actor DC	-0.006 (0.007)	-0.89	0.38	[-0.02,0.008]
Partner DC	-0.009 (0.008)	-1.22	0.23	[-0.02,0.006]
Daily Stress	0.01 (0.007)	1.83	0.07 ^τ^	[-0.001,0.03]
RSA	0.24 (0.17)	1.44	0.15	[-0.09,0.58]
Actor DC × RSA	0.0001 (0.009)	0.006	0.99	[-0.02,0.02]
Partner DC × RSA	-0.007 (0.007)	-0.97	0.34	[-0.02,0.01]
Daily Stress × RSA	-0.01 (0.008)	-1.26	0.21	[-0.02,0.006]
***Negative DC***				
Actor DC	0.01 (0.003)	3.66	<0.001**	[0.006,0.02]
Partner DC	0.01 (0.004)	2.34	0.02^∗^	[0.001,0.02]
Daily Stress	0.01 (0.006)	2.27	0.03^∗^	[0.001,0.03]
RSA	0.18 (0.10)	1.78	0.08^τ^	[-0.02,0.38]
Actor DC × RSA	-0.01 (0.004)	-2.65	0.01^∗^	[-0.02,-0.003]
Partner DC × RSA	0.002 (0.004)	0.57	0.57	[-0.006,0.01]
Daily Stress × RSA	-0.002 (0.006)	-0.25	0.80	[-0.01,0.01]

*^τ^*p* < 0.10; ^∗^*p* < 0.05; ^∗∗^*p* < 0.001*.

**FIGURE 3:**
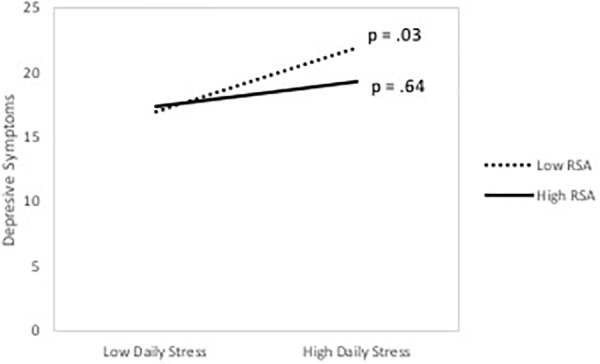
The association between daily stress and depressive symptoms as a function of respiratory sinus arrhythmia (RSA) among women. *p*-values represent the significance of the simple slopes at high (1 SD above the mean) and low (1 SD below the mean) of RSA.

**Table 3** presents the actor and partner effects as well as their interactions with actor’s RSA for women. Results indicated that there was a marginally significant actor effect of negative DC and a marginally significant partner effect of delegated DC on depressive symptoms. None of the moderation effects with RSA were significant. However, there was a significant or marginally significant daily stress by RSA interaction in three out of the four models. Simple slopes analysis indicated that among mothers with lower RSA there was a significant association between daily stress and depressive symptoms, and that the association was not significant among mothers with higher RSA. These results are depicted in **Figure 3**. To test whether the strength of the association between DC and depressive symptoms significantly differed for men and women, two-way interactions between actor and partner DC effects and gender were computed. None of the DC × gender interactions were significant, all *p*’s > 0.15. Gender differences in the moderation effects of RSA were tested using three-way interactions among gender, DC, and RSA. None of the three-way interactions were significant, all *p*’s > 0.24.

**Table 3:** Actor and partner effects of DC on depression and moderation effects of RSA for women.

	*β* (SE)	*t*	*p*	95% CI
***Supportive DC***			
Actor DC	-0.003 (0.005)	-0.64	0.53	[-0.01,0.006]
Partner DC	-0.004 (0.004)	-1.08	0.28	[-0.01,0.004]
Daily Stress	0.01 (0.007)	1.90	0.06^τ^	[-0.001,0.03]
RSA	0.23 (0.26)	-0.89	0.37	[-0.28,0.75]
Actor DC × RSA	-0.004 (0.005)	-0.79	0.43	[-0.01,0.006]
Partner DC × RSA	0.003 (0.005)	0.67	0.51	[-0.006,0.01]
Daily Stress × RSA	-0.02 (0.009)	1.90	0.06^τ^	[-0.03,0.0008]
***Delegated DC***			
Actor DC	0.004 (0.01)	-0.48	0.64	[-0.01,0.02]
Partner DC	-0.02 (0.01)	-1.94	0.06^τ^	[-0.04,0.001]
Daily Stress	0.01 (0.007)	1.86	0.07^τ^	[-0.001,0.03]
RSA	0.27 (0.23)	-0.16	0.25	[-0.20,0.75]
Actor DC × RSA	-0.003 (0.01)	0.34	0.73	[-0.02,0.02]
Partner DC × RSA	-0.001 (0.01)	-0.10	0.92	[-0.02,0.02]
Daily Stress × RSA	-0.02 (0.008)	1.90	0.04^∗^	[-0.03,-0.0004]
***Common DC***			
Actor DC	-0.005 (0.008)	-0.63	0.53	[-0.02,0.01]
Partner DC	-0.006 (0.009)	-0.71	0.48	[-0.02,0.01]
Daily Stress	0.01 (0.008)	1.91	0.06 ^τ^	[-0.001,0.03]
RSA	0.26 (0.23)	1.10	0.28	[-0.21,0.73]
Actor DC × RSA	-0.009 (0.008)	-1.16	0.25	[-0.02,0.006]
Partner DC × RSA	0.007 (0.009)	-0.71	0.48	[-0.01,0.02]
Daily Stress × RSA	-0.02 (0.008)	-2.04	0.04^∗^	[-0.04,-0.0004]
***Negative DC***			
Actor DC	0.01 (0.006)	1.84	0.07 ^τ^	[-0.001,0.02]
Partner DC	0.001 (0.005)	0.26	0.79	[-0.009,0.01]
Daily Stress	0.01 (0.007)	1.84	0.07 ^τ^	[-0.001,0.03]
RSA	0.06 (0.15)	0.43	0.66	[-0.23,0.36]
Actor DC × RSA	0.0006 (0.007)	0.09	0.92	[-0.01,0.01]
Partner DC x RSA	0.005 (0.006)	0.71	0.48	[-0.008,0.02]
Daily Stress X RSA	0.01 (0.008)	-1.46	0.15	[-0.02,0.004]

*^τ^*p* < 0.10;^∗^*p* < 0.05*.

## Discussion

The goal of this study was to evaluate whether RSA moderates the association between DC and depressive symptoms among parents of preschool children. Results indicated that actor’s RSA moderated the partner effect of delegated DC as well as the actor effect of negative DC in predicting the actor’s depressive symptoms. Men with higher RSA exhibited a stronger negative association between their female partner-rated delegated DC within the couple relationship and their own depressive symptoms, but a smaller association between their own rating of negative DC within the couple relationship and their own depressive symptoms compared to their counterparts with lower RSA. These findings suggest that men with higher RSA may possess social skills and abilities that support their DC skills.

In the current study, negative DC was the form of DC that showed the strongest association with depressive symptoms. This finding is consistent with results from prior studies (Bodenmann et al., 2011; Regan et al., 2014). Results indicated that RSA buffered the effect of negative DC on depressive symptoms among men, such that men with lower RSA displayed a larger association between negative DC and depression than participants with higher RSA. These results dovetail with findings from Diamond et al. (2011), who reported that men with lower RSA showed a stronger association between daily negative affect and daily negative spousal interaction. In another study, high RSA buffered the association between maternal depression and negative affect escalation during a mother-adolescent interaction task (Connell et al., 2011). These results are broadly consistent with the hypothesis that individuals with higher RSA might be better able to regulate their emotions in the face of negative social interactions (Thayer and Lane, 2009; Diamond et al., 2011), thereby diminishing the impact of negative DC interactions on their depressive symptoms.

In prior studies, delegated DC has been associated with both increased and decreased depression (Meier et al., 2011; Vaske et al., 2015). In the present study, RSA moderated the effects of partner-rated delegated DC on depressive symptoms. Among men, higher RSA was associated with a significant negative association between partner-rated delegated DC and depression, whereas there was a positive, but non-significant association between delegated DC and depressive symptoms when RSA was lower. Men with higher RSA thus benefited more from partner-rated delegated DC than men with lower RSA. These results are in line with those of Hopp et al. (2013) who reported that greater social support was associated with fewer depressive symptoms among individuals with higher RSA, whereas there was no association between social support and depression among individuals with lower RSA. Individuals with higher RSA may be more physiologically regulated by positive social interactions than their counterparts with lower RSA (Porges, 2003a), leading to a stronger stress-buffering effect of positive interpersonal relationships. However, it has also been suggested that delegated DC can overburden the partner in situations of high stress (Rottmann et al., 2015). Individuals with low RSA may possess less self-regulatory resources, which leaves them more easily overburdened by delegated DC, leading to an increase in depressive symptoms associated with delegated DC.

In the present study, RSA moderated the association between DC and depressive symptoms among men, but not among women. Diamond et al. (2011) reported that RSA moderated the association between negative affect and negative marital interaction among men, whereas it moderated the association between positive affect and positive interactions among women. Given that our measure of depression focused mostly on negative affect, this may explain why the findings were significant only for men. However, these gender differences should not be over-interpreted, given that the effects for men and women were not statistically different from each other. In a similar vein, RSA interacted with daily stress to predict depressive symptoms among women but not among men. The association between daily stress and depression was stronger among women with lower RSA. These results dovetail with prior results suggesting that RSA is marker of vulnerability to stress (Fabes and Eisenberg, 1997; Gouin et al., 2014).

In the DC literature, gender differences in the association between DC and psychological distress are not consistent. Although Bodenmann et al. (2011) reported that positive DC was associated with depression in women, but not in men, Chaves et al. (2018) observed that positive DC was associated with psychological distress in men but not in women. Furthermore, many studies found similar bivariate associations between DC and depression among men and women (e.g., Gabriel et al., 2016; Karademas and Roussi, 2016). In the present study, positive forms of DC were associated with depressive symptoms among men, but not among women; however, the beta coefficients between DC and depressive symptoms were in the same direction for both sexes. This suggests that a lack of statistical power may explain why these effects were not significant for women.

The association between DC and depressive symptoms is bidirectional. Greater DC can reduce depressive symptoms, but elevated depression may also erode DC (Bodenmann et al., 2004; Gana et al., 2017). Although depressive symptoms were assessed at three time points over a 12-month period in this study, there was no significant change in depressive symptoms over time for women. Therefore, the average level of depressive symptoms across the 3 times points for each partner was used in the analyses. In the context of these cross-sectional analyses, the directionality of the association between DC and depressive symptoms cannot be determined. This means that an alternative and equally plausible interpretation of these findings is that individuals with low RSA engaged in more negative DC when they experience elevated depressive symptoms compared to their counterparts with high RSA. Furthermore, individuals with high RSA may be more likely to have a partner who engaged in high delegated DC when they report low depressive symptoms. Longitudinal studies with longer follow-ups that may capture changes in both depressive symptoms and DC as they occur may help clarify the directionality of the association between these two constructs.

In this study on parenting stress of parents of preschool children, couples exhibited high levels of depressive symptoms. Indeed, the mean CES-D score was close to the clinical cut-off for risk of a major depressive disorder. Several factors may explain this high level of psychological distress. Couples rearing a child with a neurodevelopmental disorder were oversampled for this study, and these parents tend to experience higher levels of psychological distress than other parents (Olsson and Hwang, 2001). Couples who participated in this study had less education and lower household incomes than the average person in Montreal, QC, Canada (Statistics Canada, 2017). Notably, 25% of the couples lived on or below the poverty line. Higher financial strains increase risk for depression among parents of young children (Heneghan et al., 1998). Moreover, our recruitment strategy advertising a study of parenting stress may have led to a sampling bias whereby couples who experienced higher levels of parenting stress were preferentially recruited into the study. While this sampling bias limits the generalizability of the present findings, it also highlights the role of DC in the context of elevated psychological distress. Future studies should replicate these findings in samples that are more representative of the general population. Another limitation of this study is the large number of statistical tests conducted. Given the inconsistencies in the associations between the different forms of DC and depression, and the high correlations among positive DC subscales (ranging from 0.58 – 0.84), the analyses were run in multiple models to avoid multicollinearity issues. If a family wise Bonferroni correction was applied to the findings, the moderation effect of RSA on partner-rated delegated DC would no longer be statistically significant. Furthermore, the reliability of the delegated DC subscale was relatively low in this sample. These results must therefore be interpreted cautiously, and replication of these findings is paramount.

In the current paper, an APIM framework was adopted to examine both partners’ contribution to each partner’s depression. The Dyadic Coping Inventory assessed the construct of DC both from an individual perspective (e.g., I show empathy and understanding to my partner) as well as from a couple perspective (e.g., We engage in serious discussion about the problem and think through what has to be done). Rather than examining how both partners’ perception of DC is related to each partner’s depression, one could conceptualize DC as a couple-level variable shared by both partners. Using this perspective, a Common Fate Model would be an alternative way to analyze these data (Kenny and La Voie, 1985; Lederman and Kenny, 2012). Future research should evaluate whether the association between DC and depression differ when it is conceptualized as an individual or as a dyad level variable.

These findings suggest several potential future research directions. Both the polyvagal theory and the neurovisceral integration models suggest specific mechanisms through which RSA may moderate the effect of DC (e.g., reduced emotional expressiveness, impaired emotion regulation, increased sensitivity to the social context); future studies should attempt to identify the specific pathways underlying these effects. Furthermore, interventions aimed at improving DC have been associated with improvements in depressive symptoms similar to those of cognitive-behavioral therapy and interpersonal therapy (Bodenmann et al., 2008). Future research should test whether RSA may help identify individuals with depression who are more likely to benefit from a DC-focused intervention. DC has also been related to stress-related physiological processes (Meuwly et al., 2012; Kordahji et al., 2015; Gouin et al., 2016). Future studies should test whether RSA also moderates the impact of DC on physiological stress responses.

In terms of clinical implications, these results provide further evidence that promoting DC may reduce depressive symptoms (Bodenmann et al., 2008), especially among fathers of young children. Low RSA may also help identify men who are more vulnerable to the impact of negative DC on depressive symptoms. However, in order to apply these results in clinical practice, future studies should aim at establishing norms and cut-off scores of RSA that will facilitate the identification of at risk individuals.

In summary, these findings indicate that RSA moderated the association between DC and depressive symptoms. High RSA buffered the association between negative DC and depression and enhanced the association between delegated DC and depressive symptoms. These results provide further evidence that RSA modulates affective responses to social interactions. More studies are needed to examine the specific pathways through which RSA influences the DC process.

## Author Contributions

J-PG and EB designed the study. CdE and WC collected and processed the data. J-PG analyzed the data. AS and J-PG wrote the manuscript. All authors reviewed and approved the final version of the manuscript.

## Conflict of Interest Statement

The authors declare that the research was conducted in the absence of any commercial or financial relationships that could be construed as a potential conflict of interest.
